# Porcine Breed, Sex, and Production Stage Influence the Levels of Health Status Biomarkers in Saliva Samples

**DOI:** 10.3389/fvets.2019.00032

**Published:** 2019-02-14

**Authors:** Jorge Sánchez, Ana García, José María Ruiz, Ana María Montes, Juan Cabezas-Herrera, Susana Ros-Lara, Ernesto De la Cruz, Ana María Gutiérrez

**Affiliations:** ^1^Cefu S.A., Murcia, Spain; ^2^Department of Animal Medicine and Surgery, Regional Campus of International Excellence Campus Mare Nostrum, University of Murcia, Murcia, Spain; ^3^Molecular Therapy and Biomarkers Research Group, Clinical Analysis Service, University Hospital Virgen de la Arrixaca, IMIB-Arrixaca, Murcia, Spain; ^4^Department of Physical Activity and Sport, Regional Campus of International Excellence Campus Mare Nostrum, University of Murcia, Murcia, Spain

**Keywords:** pig, health status, salivary marker, variation, range values

## Abstract

In this study, the influence of several factors such as breed, sex, and production stage over the normal range values of salivary biomarkers of health status was evaluated in pigs. A total of 409 pigs of 2 different breeds (conventional Large White × Duroc and Iberian pigs) were included in the study. Animals were divided into different groups according to their sex (male or female) and the stage of the production cycle they were in (post-weaning, nursery, fattening, and finishing). The levels of an inflammatory marker, adenosine deaminase (ADA), and two acute phase proteins, C-reactive protein (CRP) and haptoglobin (Hp) were measured in saliva samples. Moreover, the total antioxidant capacity level (TAC) was quantified for the first time in porcine saliva; therefore, an analytical validation and stability analysis during storage at −80°C were also performed. Differences according to breed were observed for all the markers studied; thus, the influence of age and sex on the normal range values were studied separately for conventional and Iberian pigs. In Large White × Duroc pigs the overall median values of ADA, CRP, Hp and TAC were 282 U/L, 10.49 ng/mL, 0.88 μg/mL, and 21.73 μM Trolox equivalents, respectively. However, higher values of inflammatory marker and acute phase proteins were observed in males at the initial stages of the production cycle, while females presented higher values when they had reached sexual maturity. In Iberian pigs the overall median values observed were 585 U/L, 4.81 ng/mL, 0.63 μg/mL, and 21.21 μM Trolox equivalents for ADA, CRP, Hp, and TAC respectively with slight differences in the influence of the studied factors. Sex differences were not observed in the levels of acute phase proteins in Iberian pigs, probably due to the castration of males during the first days of life; however, ADA levels were found to be higher in male pigs at the end of the production cycle. It could be concluded that breed, sex, and production stage influence the range values of salivary markers of health status in pigs and should be taken into account to further establish reference intervals.

## Introduction

The salivary protein profiles obtained from healthy and diseased animals exhibit whole saliva's ability to reflect systemic conditions in pigs (1). In fact, the use of saliva as an analytical tool in porcine research has increased exponentially over the last years according to the PubMed database. Several studies have indicated that salivary acute phase protein (APP) concentrations can be utilized as early indicators of the health status of pigs (2, 3). This statement is of great value taking into account that some diseased pigs did not develop leucocytosis in spite of having clinical signs and pathological lesions but experienced an evident increase in APPs (4, 5). Moreover, several differences have been reported with regard to the sensitivity of different APPs for detecting disease. As an example, it has been reported that in general, bacterial infection causes a higher increase of APP than virus infections in field conditions (6). Additionally, age-related changes (7), breed and sex influences (8) and reproductive state and parity differences (9) have been identified in the plasma levels of individual APPs in pigs. Furthermore, due to its low specificity, APPs cannot be used to identify the specific etiology of the inflammation or a specific disease but the mere presence of an unspecific homeostasis disturbance: thus, the use of an APP index has been highly suggested in serum samples (10). In this context, the incorporation of other alternative markers of immune responses such as ADA, an enzyme involved in the development and maintenance of the immune system, could be of great value. ADA has been described to represent an important checkpoint to down-regulate extracellular adenosine levels and the release of inflammatory mediators in several human inflammatory disorders (11). In pigs, ADA was recently reported to be increased in local inflammatory conditions, such as arthritis or abscesses, and in gastrointestinal and respiratory disorders (12); however, its potential implication mechanism in each pathology has not been described until now.

A recent study reported that stress-induced immunosuppression affects the release of pro-inflammatory cytokines such as tumor necrosis factor-α (TNF-α) suggesting that neuroendocrine mechanisms are somehow meditated by glucocorticoids and catecholamines (13). Moreover, chronic stress has been associated with dysregulated immunity and subsequent low-grade inflammation (14). Furthermore, it has been also reported that common stressful practices during porcine production such as mixing unfamiliar animals induce oxidative stress in pigs (15). Oxidative stress may be involved in several pathological conditions, including conditions that are relevant to animal production and the general welfare of the individuals. Thus, common diseases such as pneumonia (5) and sepsis in pigs (16) have been demonstrated to involve altered redox balance. Further, research stated that the levels of antioxidants, specifically ascorbate, decline with the progression of pneumonia in pigs and become normalized after proper antimicrobial treatment (17). Moreover, the oxidant NO increases in pigs with experimentally induced enteritis (18). Many investigations have demonstrated corresponding patterns of oxidative stress between serum and saliva, suggesting that saliva contains oxidation biomarkers similar to those present in blood (19). Thus, measuring the oxidant and antioxidant status in the saliva of pigs could be a useful tool to monitor their health status; however, this method has not been explored until now.

Investigation into the sources of biological variability and interferences affecting the measurement of the analytes in question is recommended prior to the selection and preparation of reference intervals (20). The relevance of age in determining reference intervals not only in serum biochemistry and hematology but also in acute phase proteins has been widely defined in porcine in the last 20 years in conventional pigs (21–24) and minipigs (25, 26). Moreover, research has reported that the establishment of reference baseline concentrations prior to the implementation of porcine health status biomarkers is mandatory in serum samples (27). In saliva samples, analytical interferences such as stability under short or prolonged storage conditions (28) as well as daytime variations have been established for some analytes (29, 30). However, despite all the evidence reported about the usefulness of salivary biomarkers of health status in pigs, no studies have indicated the biological factors that could influence the reference range values of salivary biomarkers in the pig.

The aim of the present study was to define the possible influence of sex, breed, and production stage on the levels of salivary biomarkers of health status, specifically the inflammatory marker adenosine deaminase (ADA), the acute phase proteins CRP, and Hp, as well as the total antioxidant status (TAC) in pigs.

## Materials and Methods

### Animals and Sample Collection

Two porcine intensive farms, located in the south east of Spain and belonging to the same commercial company, were included in the study. A porcine commercial closed-cycle farm in which animals were vaccinated against enzootic pneumoniae at 7 and 28 days of age, against porcine circovirus at 28 days of age and against Aujezsky disease at 11 and 14 weeks of age approximately. The second farm was a porcine Iberian three-phase farm in which animals were vaccinated with the same protocol as the commercial farm, in addition to a third vaccination against Aujezsky disease administered at 6 months of age.

In each farm the clinical examination and saliva samplings were performed on 4 different days, one for each production stage, by the same personnel. All samples were obtained between 10 a.m., and 12 a.m., and the collection of all the samples at the different farms and herds were completed in 2 months during the spring of 2018. No outbreaks were reported in the 2 months preceding or following the sample collection.

A total of 202 and 207 animals were sampled from the commercial and Iberian pig farms, respectively. Animals in different stages of the production system (weaning, nursery, fattening, and finishing) were evaluated. The descriptive traits of the animals has been presented in Table 1, in which the primary management characteristics of the two farms, the housing conditions (space/animal), the nutritional programs (type of feeding), and the age of the animals in each stage, have been detailed. Male pigs in the Iberian farm were castrated during the first week of life, in accordance to good veterinary practice.

**Table 1:** Descriptive characteristics of the commercial Large White × Duroc and Iberian pigs including in the study.

**Breed**	**Stage**	***N***	**Sex**	**Age (days)**	**Weight (Kg)**	**Type of nutrition**	**Space per animal**
Commercial pigs	Weaning	22	Male (12) Female (10)	23	5	Dry *ad libitum*	0.45 m^2^
	Nursery	41	Male (21) Female (20)	36	10	Dry *ad libitum*	0.45 m^2^
	Fattening	54	Male (27) Female (27)	98	45	Dry *ad libitum*	0.65 m^2^
	Finishing	85	Male (42) Female (43)	150	95	Dry *ad libitum*	0.65 m^2^
Iberian pigs^*^	Weaning	36	Male (18) Female (18)	42	9	Dry *ad libitum*	0.45 m^2^
	Nursery	39	Male (20) Female (19)	75	22	Dry *ad libitum*	0.45 m^2^
	Fattening	54	Male (28) Female (26)	180	80	Liquid rationed	2 m^2^
	Finishing	78	Male (38) Female (40)	300	150	Liquid rationed	2 m^2^

* *Male pigs form Iberian farm were castrated animals*.

In the first farm, pigs were housed in pens in groups of maximum 10 animals while in the second farm pigs were housed in groups of 20 animals in the nursery period followed by groups of 8 animals in the fattening-finishing stage with a total unobstructed floor area available in line with the official standards (Directive 2008/120/EC and the Spanish Real Directive 4/2014). The animal's diet has been detailed in Table 2. All procedures involving animals were approved by the Murcia University Animal Experimentation Committee and followed the recommendations of the European Convention for the Protection of Vertebrate Animals used for Experimental and Other Scientific Purposes (Council of Europe, ETS no. 123). All methods were performed in accordance with the relevant ARRIVE guidelines and regulations. Farm owners were appropriately informed about experimental procedures prior to their implementation.

**Table 2:** Nutrient composition of the ration at the different stages of commercial Large White × Duroc and Iberian pig production.

**Breed**	**Stage**	**Water**	**Crude protein**	**Fat**	**Crude fiber**	**Ash**	**Starch**	**Ca**	***P***
Commercial pigs	Weaning^*^	12.39	16.27	5.87	2.85	3.94	43.61	0.62	0.49
	Nursery^*^	13.29	16.53	3.99	3.41	4.12	44.19	0.75	0.42
	Fattening	11.81	16.3	5.22	3.56	4.14	44.48	0.91	0.45
	Finishing	11.57	14.83	5.6	3.74	4.02	45.89	0.8	0.41
Iberian pigs	Weaning^*^	13.2	17.07	4.99	2.88	3.96	42.53	0.81	0.57
	Nursery^*^	11.6	14.62	4.15	3.96	3.87	45.36	0.92	0.44
	Fattening	10.93	13.61	4.64	3.97	3.48	46.45	0.87	0.42
	Finishing	11.34	12.96	5.31	4	3.44	46.15	0.83	0.42

Ca, calcium; P, phosphorous.

* *200 ppm tilmicosin, 200 ppm amoxicillin, and 9 ppm fenbendazole (1 ppm = 1 mg/kg)*.

Animals were subjected to a general clinical veterinary examination at the farms prior to sampling. The parameters taken into account during the examination were detailed observation of the individual animal and other pigs in the group and their environment, the general aspects of the individual animals and annotation of any clinical signs of disease or abnormal behavior. No infected lesions of the skin, diarrhea, coughing, tail bites, or lameness were registered. Sampled pigs had normal skin color and body conditions in accordance to the corresponding stage of the production system.

Saliva samples were obtained prior to the veterinary clinical examination through the introduction of a sponge of ~1 × 1 cm, clipped to a thin metal rod, in the mouth of pigs individually for 1–2 min. Sponges were placed in specific tubes (Salivette tubes, Sarstedt, Nümbrecht, Germany) and stored on ice until they were processed at the laboratory, after a period not exceeding 2 h. Saliva was obtained at the laboratory through the centrifugation of the tubes at 3,000 × g for 10 min: these were stored at −80°C until analysis in two identical aliquots, for biomarker quantification, and proteomics analysis, respectively.

### Analytical Measurements in Saliva Samples

The quantification of total adenosine deaminase (ADAt) and its isoforms 1 (ADA1) and 2 (ADA2) were performed following a previously optimized and validated commercial assay (BioSystems S.A., Barcelona, Spain) (12). The assay precision was high with an intra assay coefficient of variation (CV) lower than 5.5% and a sensitivity of 9.33 U/L.

The acute phase proteins CRP and Hp were quantified using previously developed and validated time-resolved inmunofluorometric assays (1, 2). The CV for intra and inter assay precision were below 12% and the limit of detection were 0.73 and 0.52 ng/mL for CRP and Hp, respectively.

TAC was measured in saliva samples through the ferric reducing antioxidant power (FRAP) assay (31). The method consisted in the reduction of the ferric complex tripiridil triazine (Fe3+ TPTZ) to form Fe2+ in an acidic medium. Prior to its implementation, the optimal sample dilution was calculated using a standard curve of Trolox (a water-soluble analog of vitamin E employed as a control antioxidant agent for assay calibration), ranging from 1.5 to 100 μM Trolox equivalents, and the assay was validated according to previously reported protocols (2). In brief, intra assay and inter assay precision, accuracy and limit of detection were calculated. The median CVs for intra and inter assay were below 11% (Table 3). A high accuracy was gained according to the coefficients of correlation of 0.992 observed when the linearity under dilution of two samples of high TAC levels of 84.80 and 65.83 μM Trolox equivalents, respectively were analyzed. The limit of detection of the assay was set at 0.80 μM Trolox equivalents.

**Table 3:** TAC assay validation results for the measurement of TAC concentrations in porcine saliva samples.

**Precision**	**Intra assay**			**Inter assay**		
	**X**	**SD**	**CV**	**X**	**SD**	**CV**
High TAC levels	55.62	1.33	2.60	50.03	5.58	10.99
Medium TAC levels	17.51	0.91	7.04	17.09	1.52	8.75
Low TAC levels	9.51	0.66	9.45	10.03	1.06	10.98
Total	27.55	0.96	5.02	25.71	2.72	10.24

*X, median (μM Trolox equivalent); SD, standard deviation; CV, coefficient of variation (%)*.

Biological samples were analyzed with a single determination without duplicate or replicate analysis, since the assay methods used for the quantification of markers allow acceptable validation data in accordance to the general official guidelines (32).

### Statistical Analysis

For each analyte, descriptive statistics were calculated: mean and standard deviation, median, minimum, and maximum using statistical software (GraphPad Prism 5; GraphPad software Inc., Suite, La Jolla, USA). Comparisons between groups were performed using a Mann-Whitney *U*-test using an exact sampling distribution for *U*-, or a Kruskal-Wallis test, in order to determine whether there were differences in the salivary determined analytes in relation to sex and production stage in commercial and Iberian pigs. When the Kruskal-Wallis test was used, pairwise comparisons were performed following Dunn's procedure with a Bonferroni correction for multiple comparisons, presenting adjusted *p*-values with statistical software (SPSS 19.0 software). The level of significance was set at *p* < 0.05.

## Results

### Breed Comparison on the Levels of Salivary Biomarkers Studied

General differences were observed relative to the breed in the levels of the biomarkers quantified in pig's saliva samples (Table 4).

**Table 4:** Overall statistical descriptive values of the different biomarkers quantified in saliva samples in both conventional and Iberian pigs.

	**Conventional pigs**			**Iberian pigs**			***P-value***
	**Median value**	**Percentil 25**	**Percentil 75**	**Median value**	**Percentil 25**	**Percentil 75**	
*ADAt*	282.63	195.98	407.29	585.27	446.62	791.25	0.000
*ADA1*	71.32	27.33	147.98	60.66	13.33	114.65	0.051
*ADA 2*	193.98	115.32	299.97	524.94	369.96	729.92	0.000
*CRP*	10.49	4.08	25.81	4.81	2.79	8.98	0.000
*Hp*	0.88	0.44	1.38	0.63	0.40	1.03	0.007
*TAC*	21.73	10.91	35.70	21.21	15.29	31.32	0.410

The levels of ADAt and ADA 2 were found to be higher (twice) in Iberian pigs in comparison to conventional Large White × Duroc pigs. The differences were similar across the whole production cycle, with the exception of the post-weaning stage, in which, no differences were observed. Conversely, no statistical differences were observed in the levels of ADA 1 between breeds.

Regarding acute phase protein concentrations, differences were observed for both proteins, CRP and Hp, with lower values observed in Iberian pigs in comparison to conventional pigs. The magnitudes of the differences were similar to those observed in ADAt and ADA 2, twice for CRP, and 1.34 times for Hp levels. These differences were observed at the end of the production cycle with a quicker appearance and disappearance in CRP values (from nursery to fattening) in comparison to Hp (from fattening to finishing).

On the whole, there were no differences in the levels of TAC between the two breeds studied. However, when the levels of TAC were observed taking into account the production stage, differences between breeds were observed at the post-weaning stage, with higher values in conventional pigs (2.5-fold), and at finishing stages, with higher values in Iberian pigs (2 times). Thereafter, due to these differences observed between breeds, the effect of sex and production stages on the range values of the studied markers were analyzed separately for conventional pigs and for Iberian pigs.

### Effect of Sex and Production Stage on the Levels of Salivary Markers in Conventional Large White × Duroc Pigs and Iberian Pigs

The complete descriptive statistic measures for all marker in each studied condition are reported as supplementary material for conventional Large White × Duroc pigs (Supplementary Table 1) and for Iberian pigs (Supplementary Table 2).

**Figure 1:**
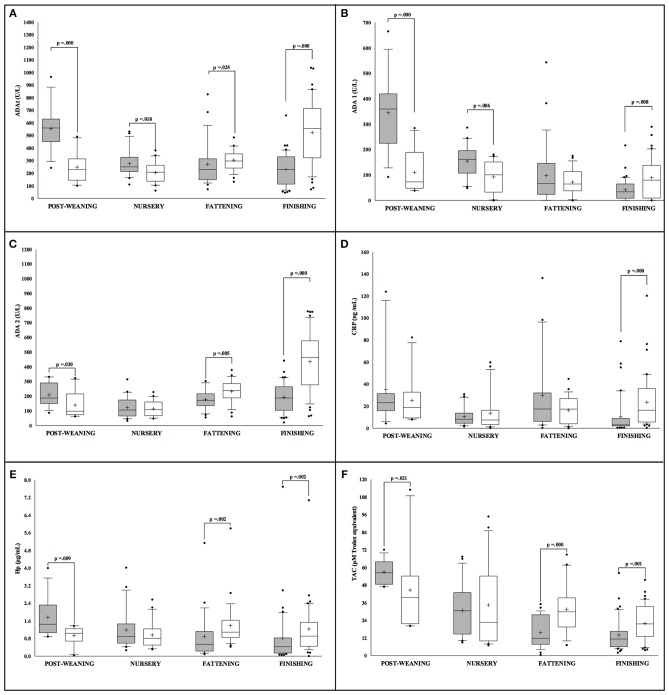
Concentration of the salivary analytes studied in conventional Large White × Duroc pigs (*n* = 202) separated according to sex (white = males and gray = females) and farming stages (X axis). Box-and-whisker plot showing median (horizontal line inside box), mean (plus symbol), 25 and 75 percentiles (edge of box), 10 and 90 percentiles (whiskers), and individual data points beyond (filled circle); significant pairwise comparisons are noted (adjusted *p*). The salivary analytes determined were ADAt **(A)**, ADA1 **(B)**, ADA2 **(C)**, CRP **(D)**, Hp **(E)** and TAC **(F)**.

The statistical analysis revealed sex differences in the levels of all the studied markers in conventional Large White × Duroc pigs (Figure 1). At the initial stages of the production cycle (post-weaning, nursery), higher values of ADAt, ADA1, ADA2, Hp, and TAC were observed in males in comparison to female pigs. On the contrary, females presented higher values of ADAt, ADA1, ADA2, CRP, Hp, and TAC in the final stages (fattening, finishing) in comparison to their male counterparts. Moreover, in male pigs, the values observed in all the markers appeared to have higher statistical significance in the post-weaning stage (*p* < 0.05). In females, no big oscillations in the levels of markers were observed in relation to the production cycle for ADA2, CRP, and Hp while higher values of ADAt, ADA2, and TAC were observed in the finishing stage (*p* < 0.05).

**Figure 2:**
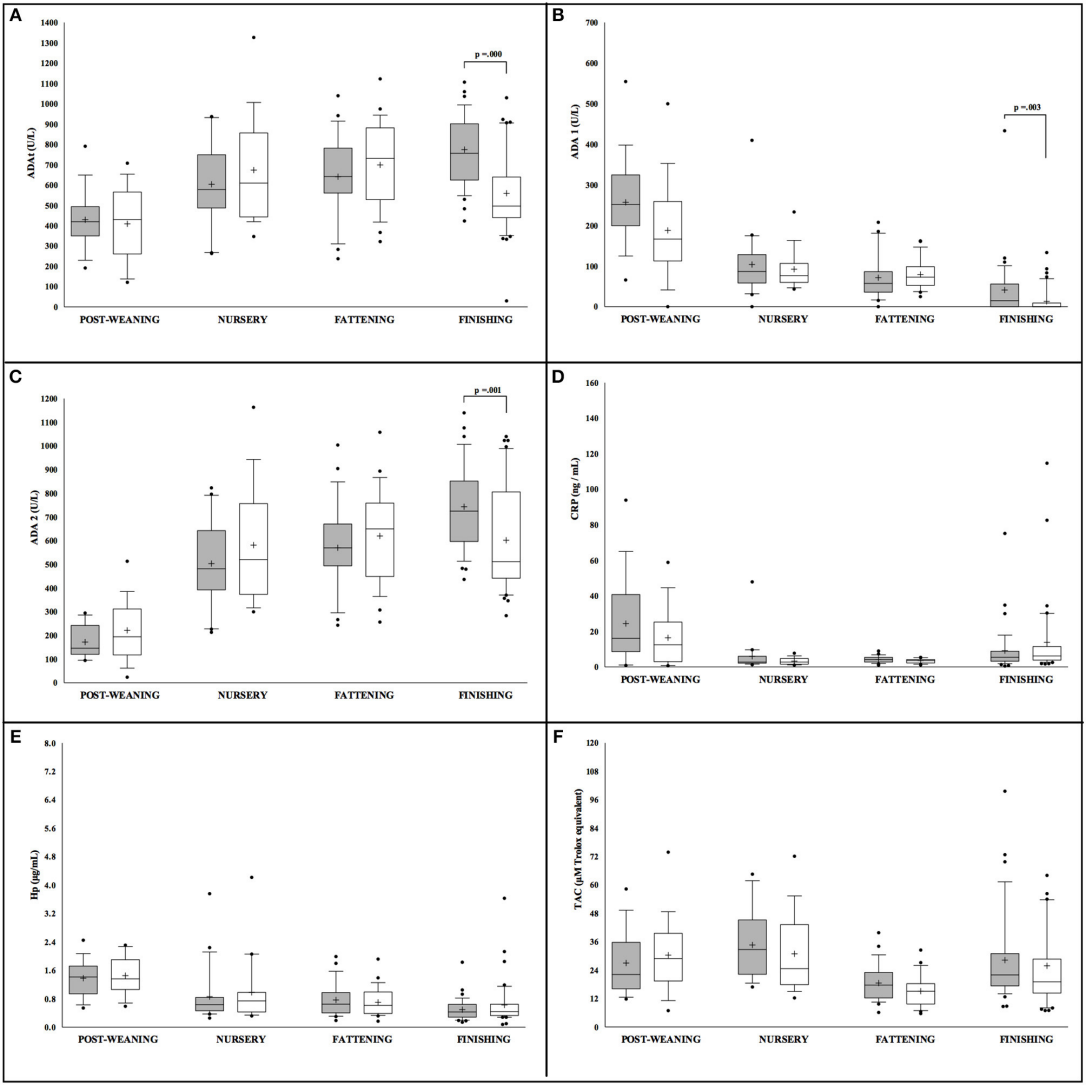
Concentration of the salivary analytes studied in Iberian pigs (*n* = 207) separated according to sex (white = males and gray = females) and farming stages (X axis). Box-and-whisker plot showing median (horizontal line inside box), mean (plus symbol), 25 and 75 percentiles (edge of box), 10 and 90 percentiles (whiskers), and individual data points beyond (filled circle); significant pairwise comparisons are noted (adjusted *p*). The salivary analytes determined were ADAt **(A)**, ADA1 **(B)**, ADA2 **(C)**, CRP **(D)**, Hp **(E)** and TAC **(F)**.

Conversely, sex differences in Iberian pigs were only observed for ADA isoforms measurements, ADAt, ADA1, and ADA2, with more statistically significant levels noted in male pigs in comparison to females but exclusively at the finishing stage (Figure 2). When the effect of production stage was analyzed in the Iberian pigs, lower levels of ADAt and ADA 2 were observed at the post-weaning stage in both males and females. On the contrary, ADA1, CRP, and Hp concentrations were lower in animals at the last stage of the production cycle (finishing), while TAC levels were constant through the whole production period with a slight decrease observed at the fattening stage.

## Discussion

In this study, the factors sex and stage of the production cycle were found to influence the baseline values of salivary markers of health status in conventional and Iberian pigs. Breed differences may be expected due to the genetic divergence between the examined breeds. Previous studies have also postulated differences in the levels of various innate immune traits between Chinese and European pig breeds (33) as well as between different European base genetic commercial lines (27). However, since the two breeds analyzed in the present study, specifically commercial European and Iberian pigs, are housing under different conditions accordingly to the official standard legal recommendations, all breed comparisons could be understood as a mix of the genetic characteristics and the effect of the specific housing conditions inherit to each breed. Sex-specific changes in the levels of health markers are predictable in adult pigs based on sexual maturity, which leads to an increase in the production of sexual hormones with implications, between other components, in the immune system (34). Moreover, the age-associated effect on the levels of salivary health markers can be expected to be similar to previous serum data on acute phase proteins (26) or pro-inflammatory cytokines (7). Our study also revealed the expected differences between animals at different stages of the production cycle, based primarily on age fluctuations (35) and different in management conditions (22).

The above-mentioned factors were studied in the following markers of health status determined in saliva samples: the acute phase proteins CRP and Hp, the enzyme ADA and the total antioxidant capacity. A total of 409 clinically healthy pigs were selected from two farms (conventional and Iberian farms) with a reported high sanitary level and no recent history of outbreaks. With regard to the possible differences in the levels of APP due to breed, sex or even stage of the production cycle, many studies have reported the influence of these in porcine serum samples over the last 20 years, as mentioned earlier. Moreover, a recent study has demonstrated the relatively stable levels of TAC during the porcine production cycle with serum samples (36). However, there is a lack of information concerning ADA range levels variation in porcine biological fluids, which is even worse in saliva samples. Thus, the results obtained in this study could be considered as basic information required prior to the establishment of salivary reference intervals in pigs and their subsequent optimal implementation as markers for health status monitoring in porcine farms.

Porcine genetic selection for high production levels has been associated with animal's increased susceptibility to environmental stressors such as pathogens, heat variations or psychological stressors (37). Differences in the response to housing environment and resistance to disease was observed when several pig breeds were compared, and it has been postulated that higher levels of immune traits, such as circulating APP, could be expected in the breeds that are less protected (33). Moreover, a more severe clinical disease was observed after an experimental infection with porcine reproductive and respiratory syndrome virus in faster growth rate lines (38). We observed higher overall values of APP in conventional Large White × Duroc pigs in comparison to Iberian pigs who may show a better protection against environmental stressors in Iberian pigs, in concordance to previous studies in which high-producing pigs showed a higher amplitude of inflammatory responses as compared to rustic animals (33). Our results could be considered contradictory to those reported recently in which high serum levels of Hp were reported in Iberian piglets in comparison to conventional crossbred piglets (39). However, the higher Hp values observed in those Iberian piglets could be a response to any environmental stressor or subclinical infection as mentioned by the authors in the report. The stated breed variations found in the levels of health status markers in our study could also be highly influenced by the different management conditions the animals were under, mainly, housing space per animal, feeding program and age of slaughter, among others, and these factors should be taken into account in future comparisons between conventional and Iberian pigs.

In contrast to the high APP values observed in conventional pigs in our stude, the levels of ADA were found to be higher in Iberian pigs in comparison to conventional Large White × Duroc pigs. These results could be related to a higher immune activation state in Iberian pigs due to the stated better protection or immune resistance of rustic animals in comparison to conventional pigs (33). ADA enzyme is likely to modulate the inflammatory processes, and ADA levels reflect monocyte/macrophage activity, with a positive correlation with CRP values in humans (40) and pigs (12). Moreover, ADA is also considered a marker of cell-mediated immunity, and ADA-deficient animals suffer from B and T lymphopaenia (41); therefore, low ADA levels could be considered an indicator of poor immune status. However, contradictory results have also been reported with respect to the association of ADA with markers of disease activity in humans (40); so, further studies should be conducted to explore the associations between ADA and APP levels in the studied breeds. To the authors' knowledge, this is the first study in which immune traits measured in saliva samples between conventional and Iberian pigs were compared, and it warrants additional studies.

When the levels of total antioxidants were compared for the two breeds studied, higher levels were observed in conventional Large White × Duroc pigs in comparison to Iberian pigs at weaning stage. The differences might be related to any management circumstances such as nutrition, since the diet differed significantly between the two breeds and the antioxidant system of the organism involves endogenous components and exogenous molecules supplied by food (36).

Differences between sexes on APP were previously observed in adult animals' serum samples. In one study, the concentration of several APPs such as alpha1- acid glycoprotein and haptoglobin was found to be higher in sows than in boars (33), while the opposite effect was observed in the case of pig-MAP (22). However, the differences between sexes were not the same in animals of different ages, showing the effect of age on APP levels. In fact, higher circulating levels of APP were observed in females at both 18 weeks and 24 weeks in comparison to males while females only had higher levels of haptoglobin at 18 weeks (33). Similarly, we recently found differences in the levels of proteins related to the immune system such as lipocalins or odorant binding proteins and also in the levels of APP, particularly CRP and Hp in adult pigs (42), with higher values observed in females in comparison to males. Sexual maturity is attained by both the male and female pig at about 5–6 months of age (43), implying the time around the growing phase of the production cycle corresponding to a period between the fattening and finishing stage in our study. We observed a high level of all the studied markers (ADAt, ADA1, ADA2, CRP, Hp, and TAC) at the end of the production cycle in females in comparison to their male conventional Large White × Duroc counterparts. Our results are in concordance with the literature in which higher levels of immune markers have been reported in saliva (42) and serum (44) samples of female in comparison to age-matched male pigs. In humans, for instance, females produce more vigorous cellular and more vigorous humoral immune reactions, are more resistant to certain infections and suffer a higher incidence of autoimmune diseases (45). However, these sex-based differences were not observed in Iberian pigs, with the exception of ADA enzyme that appeared higher in males in the finishing stage. This lack of sex differentiation on the levels of markers could be related to the castration of male Iberian pigs that could have caused a low androgenic influence on the immune system and a less aggressive behavior (46). The higher ADA values observed in male finishing Iberian pigs in comparison to females could have been influenced by other factors that should be further studied, since no explanation could be established with the actual data.

The main limitation of this study concerns the health status of the study sample, the challenge of ensuring that all animals included in the study do not suffered from any subclinical infections and therefore represent baseline conditions. However, although the differences in the levels of markers due to breed, sex, and stage of the production cycle observed in the present study, mean values for the acute phase proteins Hp and CRP were below the salivary cut-off values reported before for male pigs at the corresponding production stages (47). If those cut-off values are used for animal selection, 7 male and 1 female conventional pigs would be excluded from the study; however, this will produce no differences in the results. It should also be stated that the salivary median levels of acute phase proteins observed in porcine diseases in the field are far from the levels observed in our suspected animals (12). For salivary ADA enzyme measurements or TAC levels, no salivary cut-off values have been reported till now in pigs that could guarantee further analysis.

Establishing a reference value for biological measures is not easy due to the problem involved in discarding individuals with altered values due to subclinical diseases. However, the mean concentrations intervals described here can be considered a reasonable approach to values that can be expected in pigs from commercial farms under normal growth conditions and in the absence of disease outbreaks. The breed, sex, and stage of the production cycle of pigs should be taken into account when analyzing concentrations of health markers in saliva samples.

This report can benefit research projects and veterinarians monitoring health status markers' values in the saliva of farm pigs.

## Author Contributions

AMG: study concept and design; JR, AG, JS, AM, and SR-L: acquisition of samples and data; ED and AMG: statistical analysis; AMG and JS: interpretation of data and drafting of the manuscript; JC-H: critical revision of the manuscript for important intellectual content.

### Conflict of Interest Statement

The authors declare that the research was conducted in the absence of any commercial or financial relationships that could be construed as a potential conflict of interest.
