# Supplementary material for: Porcine Breed, Sex, and Production Stage Influence the Levels of Health Status Biomarkers in Saliva Samples
Source: Front Vet Sci. 2019 Feb 14;6:32. doi: 10.3389/fvets.2019.00032 (PMC6382678; doi:10.3389/fvets.2019.00032)
Supplement: Supplementary file 1 [file Data_Sheet_1.PDF]

Supplementary Table 1. Descriptive statistics of the values of salivary markers of health status observed in clinically healthy commercial Large White x Duroc pigs. M = male. F = female. N = number of animals. SD = Standard deviation. Min. = minimum. Max. = maximum.

		**Post-weaning**		**Nursery**		**Fattening**		**Finishing**	
	**Sex**	M	F	M	F	M	F	M	F
	**N**	12	10	21	20	27	27	42	43
**ADAt (U/L)**	Mean	553.8	248.8	278.4	205.8	271.6	304.5	230.8	522.3
	SD	175.7	122.6	106.6	82.17	174.8	82.89	129.8	260.9
	Median	559.9	229.3	251.3	208.6	230.0	298.0	231.3	555.3
	Min.	243.3	101.3	111.3	62.66	73.33	133.3	46.66	73.33
	Max.	965.2	491.3	531.9	382.0	826.6	484.6	659.3	1038
**ADA 1 (U/L)**	Mean	345.1	109.5	155.2	92.29	97.79	72.21	42.13	89.34
	SD	144.1	83.22	65.70	61.99	124.7	49.57	42.75	79.08
	Median	360.0	72.99	162.0	101.0	65.99	64.66	34.00	79.99
	Min.	92.66	38.66	48.66	0.0	0.0	0.0	0.0	-17.33
	Max.	665.3	284.6	286.6	180.6	543.3	175.3	216.6	290.0
**ADA 2 (U/L)**	Mean	208.6	139.3	123.3	113.8	177.1	232.9	193.1	436.3
	SD	80.07	88.93	71.66	53.98	66.88	76.43	99.31	199.0
	Median	187.6	97.99	106.0	109.7	172.0	238.6	188.0	464.0
	Min.	87.33	62.66	32.66	48.00	55.99	62.66	22.00	63.99
	Max.	332.0	324.0	314.6	228.6	303.3	378.6	442.6	777.9
**CRP (ng/mL)**	Mean	35.29	25.20	10.71	13.72	29.89	16.44	10.42	23.58
	SD	36.50	22.24	8.478	17.02	35.42	12.41	17.47	23.88
	Median	23.20	18.87	8.202	7.531	17.52	17.39	3.142	16.35
	Min.	4.511	8.079	1.646	0.198	0.398	0.089	0.401	0.441
	Max.	124.0	82.46	30.94	59.83	136.4	44.81	78.99	120.3
**Hp (µg/mL)**	Mean	1.763	0.935	1.188	0.961	0.888	1.391	0.820	1.232
	SD	0.892	0.407	0.930	0.600	1.048	1.043	1.280	1.188
	Median	1.450	1.040	0.895	0.810	0.540	1.090	0.425	0.910
	Min.	0.890	0.030	0.270	0.310	0.090	0.430	0.050	0.004
	Max.	4.000	1.380	4.020	2.570	5.140	5.800	7.690	7.080
**TAC (µM Trolox eq.)**	Mean	56.95	44.63	30.69	34.42	15.81	31.61	14.06	21.97
	SD	8.328	28.86	17.63	27.47	10.34	16.29	11.16	12.16
	Median	56.42	39.58	30.57	22.74	11.88	29.89	11.32	21.64
	Min.	46.92	20.20	9.033	7.049	0.591	7.190	2.081	3.786
	Max.	72.18	113.0	67.42	94.78	35.13	68.85	56.31	51.66

Supplementary Table 2. Descriptive statistics of the values of salivary markers of health status observed in clinically healthy Iberian pigs. M = male. F = female. N = number of animals. SD = Standard deviation. Min. = minimum. Max. = maximum.

		**Post-weaning**		**Nursery**		**Fattening**		**Finishing**	
	**Sex**	M	F	M	F	M	F	M	F
	**N**	18	18	20	19	28	25	38	40
**ADAt (U/L)**	Mean	429.3	409.2	603.8	673.8	641.4	699.0	774.8	559.6
	SD	142.5	178.5	205.1	260.3	207.9	204.9	169.6	204.2
	Median	419.6	429.6	578.3	609.9	642.3	731.3	756.6	496.6
	Min.	192.0	121.3	263.3	346.6	237.3	322.0	423.3	30.00
	Max.	791.3	707.9	936.6	1327	1040	1123	1107	1030
**ADA 1 (U/L)**	Mean	257.2	187.8	103.8	92.45	71.37	79.25	41.05	13.08
	SD	110.4	119.0	84.15	49.00	57.15	36.36	74.58	29.91
	Median	251.6	166.7	86.66	76.66	57.33	72.66	15.00	0.0
	Min.	65.99	0.0	0.0	43.33	0.0	24.66	0.0	0.0
	Max.	554.6	500.0	410.0	233.3	208.0	162.0	433.3	133.3
**ADA 2 (U/L)**	Mean	172.2	221.5	503.3	581.3	570.3	619.8	743.2	601.9
	SD	70.18	128.3	181.0	239.6	184.7	200.8	181.4	219.6
	Median	146.0	194.3	481.6	519.9	569.9	649.9	724.9	511.6
	Min.	94.66	24.00	213.3	300.0	243.3	256.6	436.6	283.3
	Max.	294.6	513.3	823.3	1163	1004	1058	1140	1040
**CRP (ng/mL)**	Mean	24.43	16.44	6.056	3.297	4.277	3.438	9.185	13.88
	SD	24.54	15.84	10.42	1.984	1.898	1.238	13.08	21.80
	Median	16.12	12.51	2.743	2.747	4.196	3.578	5.435	6.209
	Min.	0.900	0.700	1.259	1.068	1.067	1.100	0.519	1.707
	Max.	93.90	58.91	47.90	7.724	9.000	5.411	75.18	114.7
**Hp (µg/mL)**	Mean	1.382	1.453	0.861	0.981	0.767	0.701	0.494	0.628
	SD	0.514	0.526	0.796	0.916	0.457	0.402	0.312	0.624
	Median	1.415	1.360	0.635	0.740	0.650	0.615	0.430	0.440
	Min.	0.540	0.590	0.260	0.320	0.190	0.170	0.140	0.080
	Max.	2.450	2.310	3.760	4.220	1.990	1.920	1.830	3.630
**TAC (µM Trolox eq.)**	Mean	27.01	30.36	34.64	30.90	18.55	15.14	28.27	25.87
	SD	13.76	16.04	14.23	15.90	7.749	6.666	19.06	22.72
	Median	22.17	28.90	32.71	24.68	17.68	15.10	22.02	19.10
	Min.	11.80	6.904	16.89	12.27	6.092	5.700	8.667	6.954
	Max.	58.30	73.85	64.55	72.16	39.80	32.49	99.62	138.8
